# Antibody-Secreting Cells To Diagnose Mycobacterium tuberculosis Infection in Children in Pakistan

**DOI:** 10.1128/mSphere.00632-19

**Published:** 2020-02-05

**Authors:** Najeeha Talat Iqbal, Kumail Ahmed, Farah N. Qamar, Fariha Shaheen, Aisha Mehnaz, Fehmina Arif, Amna Afzal Saeed, Aneeq Muhammad Yousuf, Syeda Fatima Raza, Shazia Sultana, Shahida Mumtaz Qureshi, Shakil Ahmad Siddiqi, Eric Houpt, Tania Thomas

**Affiliations:** a Department of Paediatrics, Aga Khan University, Karachi, Pakistan; b Department of Biological and Biomedical Sciences, Aga Khan University, Karachi, Pakistan; c Paediatric Ward I, Civil Hospital Karachi, Karachi, Pakistan; d School of Medicine, Aga Khan University, Karachi, Pakistan; e Sindh Government Hospital Korangi, Karachi, Pakistan; f Division of Infectious Diseases and International Health, University of Virginia, Charlottesville, Virginia, USA; Albert Einstein College of Medicine

**Keywords:** antibody-secreting cells, biomarkers, tuberculosis

## Abstract

Reliance on microbiologic methods to diagnose Mycobacterium tuberculosis infection is a suboptimal approach for children due in part to the paucibacillary nature of the disease. A blood-based biomarker assay, such as the mycobacterial-antibody-secreting cell (MASC) assay, could be a major advance for the field of study of pediatric tuberculosis (TB). Children <15 years of age with clinical concern for TB and age-matched children with no concern for TB were enrolled from outpatient clinics in Karachi, Pakistan. MASC, ferritin, and C-reactive protein (CRP) assays were performed, and results were compared among cases and controls, as well as among children with a case definition of “confirmed TB,” “probable TB,” or “possible TB.” MASC responses were significantly higher among children with TB than among controls (0.41 optical density [OD] versus 0.28 OD, respectively, *P* < 0.001), and the differences were largely driven by the data from children with confirmed TB (*P* = 0.002). Ferritin and CRP values were significantly higher among those with confirmed TB than among those with the other disease states and controls (*P* = 0.004 and *P* = 0.019, respectively). The use of the MASC assay as a blood-based biomarker for TB disease shows some promise among children with microbiologically confirmed disease; however, the performance characteristics for the majority of young children with unconfirmed TB were suboptimal in this cohort.

**IMPORTANCE** Tuberculosis (TB) in children represents a missed opportunity for diagnosis and preventive therapy. The magnitude or burden of disease in children is not fully understood due to our limitations with respect to exploring sensitive diagnostic algorithms. In a setting of TB endemicity in Pakistan, we carried out a proof-of-concept study to evaluate for the first time the performance of B cell analyses by the use of well-defined diagnostic criteria and NIH consensus guidelines as “culture-confirmed,” “probable,” and “possible” TB groups. In contrast to detection of serum antibody, we focused on mycobacterial-antibody-secreting cell (MASC) detection as a marker of active disease in children with a strong suspicion of TB. Further work exploring a larger panel of inflammatory biomarkers and enrichment of B cells with the objective of increasing the sensitivity of the current MASC assay would lead to the development of a field-friendly assay for timely diagnosis of childhood TB.

## Abstract (precis)

Tuberculosis (TB) in children represents a missed opportunity for diagnosis and preventive therapy. The magnitude or burden of disease in children is not fully understood due to our limitations with respect to exploring sensitive diagnostic algorithms. In a setting of TB endemicity in Pakistan, we carried out a proof-of-concept study to evaluate for the first time the performance of B cell analyses by the use of well-defined diagnostic criteria and NIH consensus guidelines as “culture-confirmed,” “probable,” and “possible” TB groups. In contrast to detection of serum antibody, we focused on mycobacterial-antibody-secreting cell (MASC) detection as a marker of active disease in children with a strong suspicion of TB. Further work exploring a larger panel of inflammatory biomarkers and enrichment of B cells with the objective of increasing the sensitivity of the current MASC assay would lead to the development of a field-friendly assay for timely diagnosis of childhood TB.

## INTRODUCTION

Pakistan is among the top five high-burden countries for tuberculosis (TB) (1). It is recognized that a large gap exists between the estimated number of actual incident cases and the number of cases notified to the National TB Program (NTP), which likely reflects underdiagnosis and/or underreporting of cases. Underdetection is especially relevant for children with TB due to challenges in confirming the diagnosis (2).

Currently, there is no gold standard test available for the accurate diagnosis of TB in children. The mainstays of diagnosis for adults, including the detection of Mycobacterium tuberculosis by culture/molecular methods or the detection of acid-fast bacilli (AFB) by smear microscopy, have suboptimal performance in children due in part to the paucibacillary nature of the disease. Availability of alternative testing methods that do not rely upon detection of the actual organism would be a tremendous advance in the field of pediatric TB (3). Such biomarkers are urgently needed as we move toward global goals for TB elimination.

**FIG 1:**
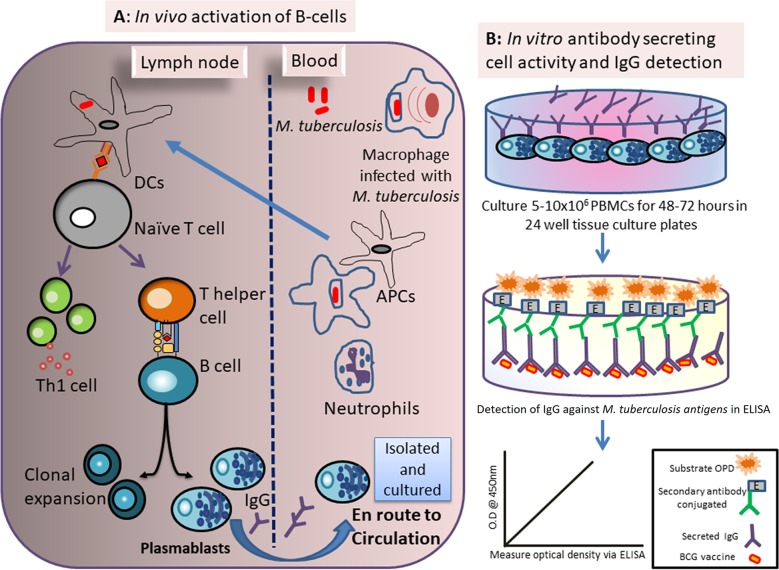
Principles of the MASC assay. (A) After Mycobacterium tuberculosis infects a macrophage, antigen-presenting cells (APCs) such as dendritic cells (DCs) migrate into lymph nodes to present M. tuberculosis antigens to naive T cells, prompting differentiation into Th1 and Th2 subsets. Activated T helper cells then activate B cells by binding CD40L to CD40 molecules present on B cells. These activated B cells can differentiate into antibody-secreting cells such as plasmablasts as well as memory B cells. Plasmablasts temporarily circulate in the bloodstream, as shown by the blue arrow, and can be collected in peripheral blood compartment. (B) In the ALS assay, peripheral blood mononuclear cells (PBMCs) containing plasmablasts are cultured in 24-well tissue culture plate for secretion of IgG antibodies. These antibodies are collected in culture supernatant for future quantification using an ELISA with bacillus Calmette-Guérin (BCG) vaccine or M. tuberculosis-specific antigens as the coating antigens. OPD, o-phenylenediamine dihydrochloride.

The mycobacterial-antibody-secreting cell (MASC) assay is a blood-based host biomarker that measures ongoing immune activation to TB by harvesting peripheral blood mononuclear cells (PBMCs) and culturing them without antigenic stimulation. IgG antibodies secreted into the culture supernatants were measured by enzyme-linked immunosorbent assay (ELISA) using the bacillus Calmette-Guérin (Mycobacterium bovis BCG) vaccine as the coating antigen as shown in Fig. 1. This assay has performed well as a TB diagnostic among adults from Bangladesh (4, 5), Ethiopia (6), and Tanzania (7). Published pediatric evaluations are limited to cohorts from Bangladesh, where mixed results have been seen. Initial studies suggested that the assay outperformed various clinical scoring systems in differentiating hospitalized children with TB from other causes of illness with 91% sensitivity and 87% specificity compared to a composite clinical reference standard (8). However, the level of performance was reduced among young children admitted with pneumonia in the setting of severe acute malnutrition (sensitivity of 67% and specificity of 51%) (9). The assay has not been evaluated among the members of an ambulatory pediatric population. Thus, we sought to validate the performance of the MASC assay as well as of other acute-phase reactants such as C-reactive protein (CRP) and ferritin (24) among children who were undergoing outpatient evaluation for pulmonary TB in Karachi, Pakistan, in comparison to healthy control children.

## RESULTS

### Characteristics of study subjects.

**FIG 2:**
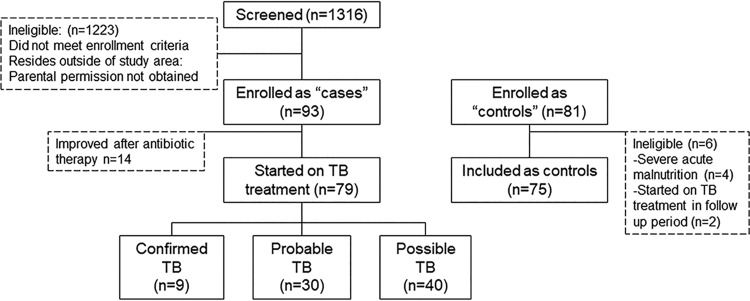
Study recruitment flow chart. IgG secretion is displayed as MASC responses in optical densities in study groups whose members were classified by disease category as follows: (i) confirmed TB; (ii) probable TB; (iii) possible TB; (iv) controls. The MASC response was assessed after 48 and 72 h. The threshold for a positive assay was an OD of 0.35.

Between April and July 2015, 79 children were enrolled and started on TB treatment; all had responded to TB treatment at the follow-up visit. A total of 75 age-matched controls were included (Fig. 2); 2 controls were excluded from the analyses as they developed TB during the follow-up period. The baseline characteristics of the study subjects are displayed in Table 1. Notable differences are seen in the overall nutritional status—children with TB were significantly more malnourished than the controls, which, in part, reflects the exclusion criterion of severe acute malnutrition among controls.

**TABLE 1:** Baseline characteristics of participants^*a*^

Parameter	Value(s)		
	Cases, *n* = 79	Controls, *n* = 75	*P*
Age, mean yrs ± SD			
All	7.8 ± 3.3	7.7 ± 3.4	ns
<5 yrs (% of total)	18 (23)	18 (24)	ns
≥5 yrs (% of total)	61 (77)	57 (76)	ns
Female, *n* (%)	44 (55.7)	45 (60)	ns
Weight (mean kg ± SD)	18.2 ± 7.2	20.6 ± 7.6	0.052
Height (mean cm ± SD)	111.2 ± 23.9	116.6 ± 19.5	ns
MUAC (mean cm ± SD)	15.2 ± 2.2	16.5 ± 2.3	<0.0001
WAZ Z-score (mean ± SD among participants <10 yrs of age)^*b*^	−2.3 ± 1.2	−0.6 ± 4.2	0.003
WHZ Z-score (mean ± SD among participants <5 yrs of age)^*c*^	−1.2 ± 1.9	−0.7 ± 0.9	ns
HAZ Z-score (mean ± SD)	−2.2 ± 1.6	−0.4 ± 5.7	0.007
BAZ Z-score (mean ± SD)	−1.5 ± 1.7	−1.0 ± 0.9	0.030

a ns, nonsignificant; MUAC, mean upper arm circumference; BMI, body mass index; WAZ, weight for age; WHZ, weight for height; HAZ, height for age; BAZ, BMI for age. Values represent results of comparisons of data from the TB-confirmed group performed using the chi-square test for proportions and the *t* test for mean values.

b Sample sizes of 63 and 56, respectively.

c Sample sizes of 18 and 15, respectively.

Table 2 compares the clinical characteristics of children with TB. Among them, 9 (11%) were confirmed by GeneXpert MTB/RIF testing, 30 (38%) were categorized as having “probable TB,” and 40 (51%) were categorized as having “possible TB.” Children categorized as having “confirmed TB” were significantly older than those in the “probable TB” and “possible TB” groups (*P* < 0.01) and were more likely to be female (89% versus 53% and 50%, respectively, *P* = 0.094). Positive tuberculin skin test (TST) reactions were not common, occurring in only 10% of children with TB and 0% of controls. There were no significant differences seen in the measurements of malnutrition among children in the confirmed TB, probable TB, and possible TB groups.

**TABLE 2:** Clinical characteristics of children with TB by classification category^*a*^

Characteristic	Values		
	Confirmed TB (*n* = 9)	Probable TB (*n* = 30)	Possible TB (*n* = 40)
Age, mean yrs ± SD			
All	11.4 ± 3.8^*b*^	7.4 ± 3.2^*b*^	7.3 ± 2.8^*b*^
<5 yrs	1 (11)	9 (30)	8 (20)
≥5 yrs	8 (89)	21 (70)	32 (80)
Female	8 (89)	16 (53)	20 (50)
Persistent cough	9 (100)	29 (97)	37 (93)
Persistent fever	9 (100)	29 (97)	37 (93)
Weight loss/failure to gain weight	2 (22)	8 (27)	9 (23)
Reduced playfulness	7 (78)	20 (66.7)	24 (60)
Known TB exposure	5 (56)	22 (73.3)	23 (58)
Positive TST^*c*^	3 (33)	3 (10)	2 (5)
BCG scar present	8 (89)	16 (53)	22 (55)
Abnormal chest radiograph	6 (67)	13 (43.3)	0 (0)
MUAC (cm) (mean ± SD)	16.5 ± 3.1	15.2 ± 1.9	14.8 ± 2.03
BAZ Z-score (mean ± SD)	−2.29 ± 1.92	−1.58 ± 1.74	−1.26 ± 1.65
Undernourished (BAZ score less than −2)	5 (56)	13 (43.3)	13 (33)
HAZ Z-score (mean ± SD)	−1.98 ± 1.39	−2.13 ± 1.75	−2.31 ± 1.54
Stunted (HAZ score less than −2)	4 (44.4)	14 (46.7)	23 (58)

a Values represent number (percent) unless otherwise indicated and represent results of comparisons to the confirmed TB group performed using ANOVA for means and Kruskal-Wallis H test for medians.

b *P* = 0.001.

c A TST measurement was defined as representing a positive result if it was ≥10 mm in any child or ≥5 mm in any child with severe malnutrition.

### Kinetics of IgG secretion.

To determine the optimal assay methodology, we compared IgG responses after 48 and 72 h of incubation (see Fig. S1 in the supplemental material). Although the IgG responses were higher after longer incubation, the differences were not statistically significant among the participants from any disease category. The proportions of positive assays also did not significantly differ by incubation time; among the seven confirmed TB participants (78%) who had a positive MASC response, all had mounted this response after 48 h of incubation. Among the probable TB participants, the 72-h incubation method yielded one additional positive response (14/27 positive responses after 72 h compared to 13/27 after 48 h of incubation; Fig. S1). To preserve a shorter turnaround time for the assay, 48-h IgG responses were considered the optimal response.

10.1128/mSphere.00632-19.1 FIG S1 Kinetics of antibody secretion based on incubation time. IgG secretion data are displayed as MASC responses quantified by optical density in study groups. (a) Confirmed TB. (b) Probable TB. (c) Possible TB. (d) Controls. The MASC response was assessed at 48 to 72 h. Download FIG S1, TIF file, 0.2 MB. Copyright © 2020 Iqbal et al. 2020 Iqbal et al. This content is distributed under the terms of the Creative Commons Attribution 4.0 International license.

### MASC performance.

**FIG 3:**
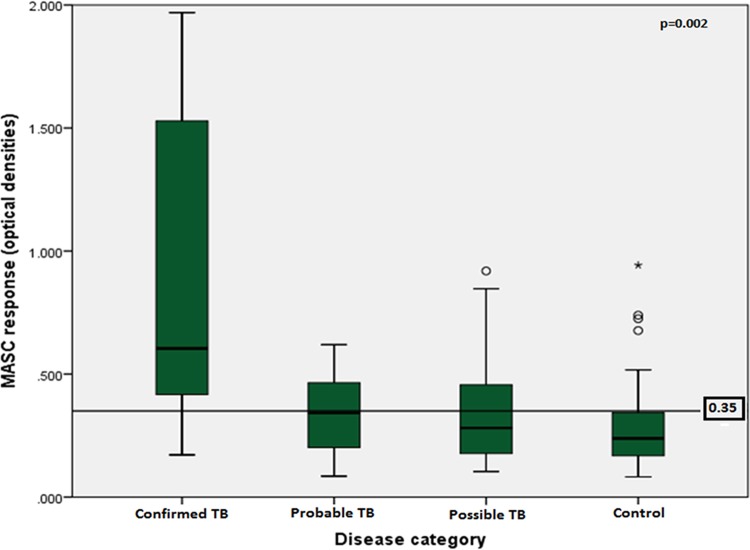
MASC responses by disease category at 48 h. Box plots demonstrate the median and interquartile values, and whiskers indicate the range of MASC responses (in optical densities) after 48 h of peripheral blood mononuclear cell culture, based on disease category. The Kruskal-Wallis test was used to compare medians across groups (*P* = 0.002).

MASC responses were significantly higher among children with TB than among controls (0.41 optical density [OD] versus 0.28 OD, respectively, *P* < 0.001), and the differences were largely driven by data representing children with confirmed TB (*P* = 0.002); Fig. 3 depicts a comparison of MASC responses by disease category. Overall, 47% of children with TB mounted positive MASC responses, including 7/9 (78%) of children with microbiologically confirmed TB, 14/30 (47%) of children with probable TB, and 16/39 (41%) of children with possible TB. Among control children, there were 16/72 (22%) who mounted positive MASC responses above the 0.35 OD threshold. Analyzing the confirmed TB cases and controls, the sensitivity and specificity were each 78%; this dropped to a sensitivity of 53% with the inclusion of children categorized as having probable and possible TB. Overall, the positive predictive value of the MASC assay was 69% using the 0.35 value as the threshold for a positive test.

The effects of key clinical characteristics on MASC responses were assessed. Among the members of the entire cohort, linear regression analysis did not demonstrate any significant effects of age (*P* = 0.10), nutritional status (height for age [HAZ], *P* = 0.23; body mass index [BMI] for age [BAZ], *P* = 0.19), or gender (*P* = 0.82) on MASC responses. Comparing participants who were <5 years of age, no significant differences in median MASC responses were seen (*P* = 0.869) between children with TB and controls; however, the same did not hold true for cases and controls ≥5 years of age (*P* = 0.004). Differences among children ≥5 years of age were driven by higher median MASC titers in the “TB confirmed” subgroup than in the other groups (*P* = 0.002). Among the children in the “TB confirmed” group, age adjustment significantly decreased the MASC responses at 48 and 72 h (*P* = 0.01; see Table S1 in the supplemental material). The potential for confounding of MASC results by age was examined through regression analysis in a multivariable model (Table S2). Evaluating children with TB only, microbiological confirmation was significantly associated with MASC results (*P* < 0.0001) but age (*P* = 0.885), HAZ (*P* = 0.810), BAZ (*P* = 0.979), and gender (*P* = 0.979) were not. Having microbiologically confirmed TB was associated with a 0.50 OD increase in MASC responses.

10.1128/mSphere.00632-19.3 TABLE S1 MASC response in children below and above 5 years of age. Data presented as means ± SD (*n*), and a *t* test was applied to compare cases to controls. Download Table S1, DOCX file, 0.01 MB. Copyright © 2020 Iqbal et al. 2020 Iqbal et al. This content is distributed under the terms of the Creative Commons Attribution 4.0 International license.

10.1128/mSphere.00632-19.4 TABLE S2 Association of age, gender, HAZ, and BAZ with MASC evaluated using a linear regression model. Download Table S2, DOCX file, 0.01 MB. Copyright © 2020 Iqbal et al. 2020 Iqbal et al. This content is distributed under the terms of the Creative Commons Attribution 4.0 International license.

The “micro-ALS” (antibody in lymphocyte supernatant) method (5) was performed for a subset of participants (*n* = 18 TB cases and *n* = 26 controls). Overall, children with TB mounted significantly higher MASC responses than the control children (0.51 OD versus 0.24 OD, *P* = 0.002). The working cutoff value was calculated as 0.51 OD, resulting in identification of 7 children with TB (39%) (3 with confirmed TB; 2 with probable TB; and 2 with possible TB) who demonstrated a positive result by this method. The specificity was 96%. The micro-ALS MASC responses are depicted by methodology and disease group in Fig. S2.

10.1128/mSphere.00632-19.2 FIG S2 Comparison of MASC results obtained using micro-ALS and the standard method. Data are from a subset of participants with paired data available. MASC responses (quantified as optical density) are displayed by incubation time (E. Akhtar, A. Mily, A. Haq, A. Al-Mahmud, et al., Nutr J 15:75, 2016, https://doi.org/10.1186/s12937-016-0194-5). The micro-ALS method included culturing 10 million PBMCs/ml for 24 h; the standard method included culturing 5 million PBMCs/ml for 48 h. A working cutoff of 0.51 OD was used for the micro-ALS method; a cutoff of 0.35 OD was used for the standard method. Download FIG S2, TIF file, 0.2 MB. Copyright © 2020 Iqbal et al. 2020 Iqbal et al. This content is distributed under the terms of the Creative Commons Attribution 4.0 International license.

### Inflammatory marker responses.

**FIG 4:**
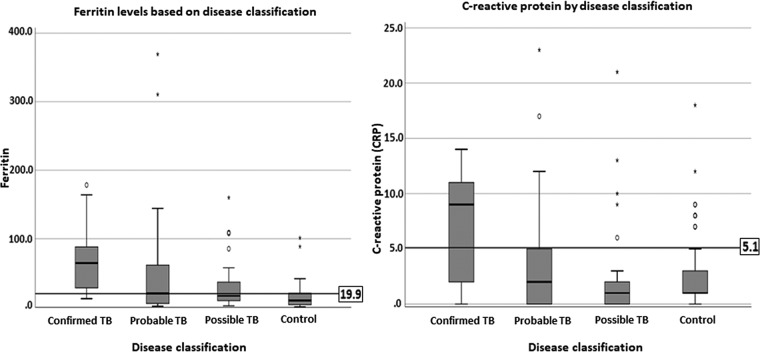
Inflammatory marker responses by disease category. Box plots demonstrate the median and interquartile values, and whiskers indicate the ranges of ferritin (A) and C-reactive protein (B) responses.

Ferritin and C-reactive protein (CRP) were evaluated as biomarkers of TB disease by comparing median values among groups, as displayed in Fig. 4. Ferritin values were significantly higher among those with confirmed TB than among those with other disease states and controls (*P* = 0.004). Linear regression analysis found that age was associated with increased ferritin levels (*P* = 0.04); every 1 month increase in age was associated with a 0.3 ng/ml increase in ferritin response. HAZ (*P* = 0.95), BAZ (*P* = 0.06), and gender (*P* = 0.97) did not have significant effects on ferritin responses.

CRP levels were also higher among participants with confirmed TB (*P* = 0.019). Linear regression analysis revealed that lower BAZ levels were associated with increased CRP levels (*P* = 0.03); every Z-score decrease in BMI for age by a value of 1 was associated with a 0.7-mg/liter-higher CRP value. Age (*P* = 0.73), HAZ (*P* = 0.56), and gender (*P* = 0.28) were not associated with CRP responses. A significant positive correlation between CRP and MASC responses was observed for the whole cohort but not for individual groups (Table S3).

10.1128/mSphere.00632-19.5 TABLE S3 Correlation of CRP with MASC assay. Download Table S3, DOCX file, 0.01 MB. Copyright © 2020 Iqbal et al. 2020 Iqbal et al. This content is distributed under the terms of the Creative Commons Attribution 4.0 International license.

### ROC analyses for MASC, ferritin, and CRP.

**FIG 5:**
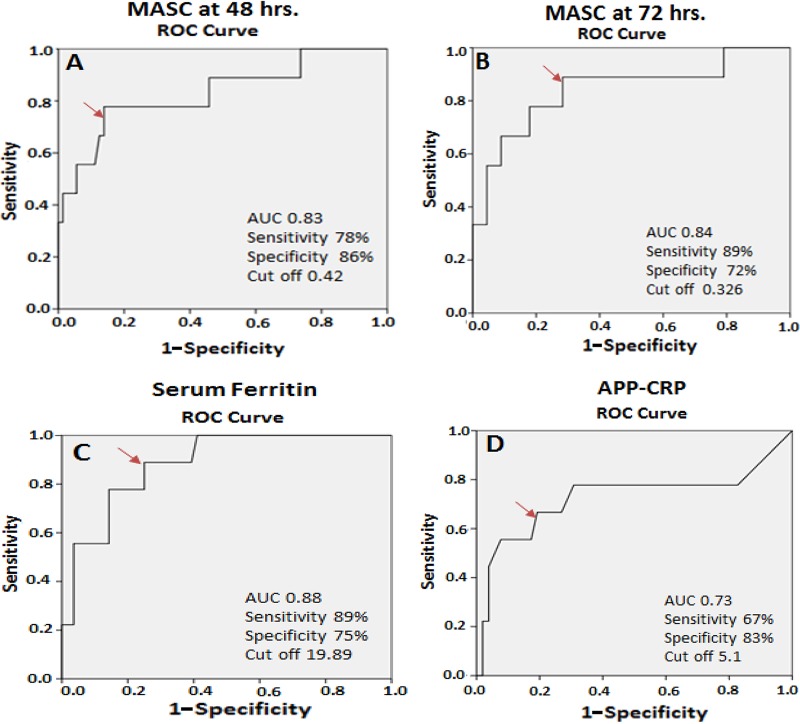
Discriminatory biomarkers of pediatric tuberculosis. ROC curves show the sensitivity and specificity cutoffs of biomarkers in comparisons between confirmed TB and healthy controls. (A) MASC at 48 h. (B) MASC at 72 h. (C) Ferritin. (D) APP-CRP (acute-phase protein–C-reactive protein). The cutoff point of each biomarker is shown on the plot; red arrows indicate the level of sensitivity for each biomarker.

The receiver operating characteristic (ROC) analysis was performed to identify cutoff values that maximized the sensitivity and specificity of the MASC, ferritin, and CRP assays. Participants with confirmed TB (*n* = 9) represented the disease state, and healthy participants (*n* = 75) represented the control state (Fig. 5A). The ROC curve data were good for the MASC (area under the curve [AUC] value of 0.83) and ferritin (AUC of 0.88) assays and fair for the CRP (AUC of 0.73) assay. For the MASC assay, the optimal cutoff was calculated at 0.41 OD, yielding sensitivity of 78% and specificity of 86%. The ferritin assay demonstrated sensitivity of 89% and specificity of 75% using a cutoff of 19.9 ng/ml. The C-reactive protein was less discriminatory; a cutoff of 5.1 mg/liter yielded sensitivity of 66.67% and specificity of 83.02%. The performance levels of all three assays were found to be further reduced in comparisons of all TB cases to healthy controls (data not shown).

## DISCUSSION

This prospective age-matched case-control study evaluated the performance of the novel MASC assay as a host biomarker of TB disease activity among children receiving ambulatory care within Karachi, Pakistan. We found that MASC response levels were greater among the children diagnosed with TB than among the control children (*P* < 0.001). These findings were driven by high responses among those with confirmed TB and were not affected by age, gender, or nutritional status.

The sensitivity of the MASC assay was 78% among children with microbiologically confirmed TB, a level which exceeds the optimal diagnostic sensitivity standard for childhood intrathoracic TB (≥66%) set forth by the World Health Organization’s target product profile for a non-sputum-based TB biomarker (11). And yet, children with clinically diagnosed TB demonstrated significantly lower responses (*P* < 0.0001), reducing the sensitivity to 47% among the entire cohort.

Initial reports of this assay in childhood TB suggested a correlation with bacillary burden; serial measurements demonstrated reductions in ALS titers which correlated well with clinical treatment response among culture-confirmed TB cases (8, 12). However, subsequent reports from studies performed with young malnourished children suggested that this assay did not outperform clinical scoring systems for diagnosis of disease in children with TB such as the Kenneth-Jones and WHO scoring criteria (9).

Given that our clinically diagnosed children demonstrated symptom resolution and improvement in anthropometrics at the follow-up visit, we feel reassured that confounding caused by non-TB diagnoses has been minimized (see Table S4 in the supplemental material).

10.1128/mSphere.00632-19.6 TABLE S4 Follow-up of cases for anthropometrics at 4 to 6 weeks. Download Table S4, DOCX file, 0.01 MB. Copyright © 2020 Iqbal et al. 2020 Iqbal et al. This content is distributed under the terms of the Creative Commons Attribution 4.0 International license.

The differences noted between microbiologically and clinically diagnosed children may represent a reflection of the bacillary burden. A greater bioburden of disease imparts increased antigenic stimulation of B cells, which may have subsequent effects on plasmablast frequency and activity. This was demonstrated by Ashenafi et al. in testing this assay among adults from Ethiopia; using flow cytometry, they demonstrated a greater proportion of plasmablasts (CD3^−^ CD19^+^ CD20^−^ CD27^high^ CD38^high^) circulating among participants with active TB than among asymptomatic participants. Additionally, they found a positive correlation between plasmablast quantity and pathogen-specific antibody secretion (6). To leverage this response, we performed the micro-ALS method on a subset of participants and indeed measured a greater MASC response by using the higher concentration of plasmablasts (i.e., 10 million PBMCs rather than 5 million PBMCs) (5). The shorter incubation time (24 h) allowed an improved turnaround time, approximating the “rapid” parameters desired in the target product profile of a non-sputum-based TB biomarker (11). Studies by our group using additional optimization methods, including sorting and testing of CD19^+^ cell secretions, are underway.

Within the members of the control population, 22% demonstrated a positive MASC result yielding a specificity value of 78%. While this is lower than the 87% specificity initially reported in children from Bangladesh, it is much higher than the 51% specificity subsequently found among children <5 years of age admitted with radiographic pneumonia and severe acute malnutrition (8, 9). We deemed these to be false-positive results for active TB because the control children in our study had no reported risk factors, symptoms, or clinical findings consistent with TB. A recent TB exposure could provide similar antigenic stimulation of plasmablasts, leading to antimycobacterial antibody detection through the use of the MASC assay (13). Although we aimed to exclude children with recent exposure to an infectious TB source, we cannot ignore this possibility in our setting of TB endemicity. It is unlikely that these results reflect cross-reaction from prior BCG vaccine administration; the BCG vaccine was used as the coating antigen for the ELISA, but no participants had received the vaccine in the 8 weeks prior to harvesting of PBMCs (14, 15). Alternatively, the positive MASC results could reflect other, nonspecific events of antibody reactivity. As a coating antigen, the BCG vaccine is thought to have a variety of epitopes to capture secreted antibodies; these may include antigens preferentially expressed by actively multiplying or semidormant bacilli (16, 17). Investigations by Rekha et al. (5) have examined the utility of using other mycobacterium-specific antigens for the ELISA among adults, such as lipoarabinomannan and those found in the RD-1 region of M. tuberculosis complex. There was no single antigen that outperformed BCG; simultaneous assessments of BCG with seven other TB antigens did not significantly increase the assay’s specificity (5). The rationale of using BCG in our experiments was based on the abundance of multiple epitopes of protein Ag85A, a glycolipid lipoarabinomannan (LAM) that not only activates B cell but also enhances CD4^+^ T cell responses (18). For standardization of our assays, we also used the same BCG from Japan as that used in earlier studies. Exploration of other candidate TB antigens is an important step that may improve the performance of the MASC assay.

Participants with microbiologically confirmed TB were significantly older than the children with clinically diagnosed TB (*P* = 0.001). This was most likely a reflection of clinical feasibility; older children are more likely to be able to produce a sputum specimen and may be more commonly offered GeneXpert testing in the ambulatory setting, where gastric aspirates are not performed. IgG responses are known to mature with age, reaching adult levels after 6 years of age (19), and yet we did not find an association with age and MASC response in our adjusted regression analysis. A greater volume of blood was obtained from older children. However, this was unlikely to affect MASC responses because a standardized concentration of PBMCs was used for all cell cultures (5 or 10 million PBMCs/ml). The purpose of obtaining a greater blood volume from older children was to account for the age-related reduction in PBMCs as well as in CD19^+^ cell concentrations (20). In its current format, this assay requires PBMC isolation and tissue culture facilities which may not be available in many high-burden TB settings.

Few children in this study had a positive tuberculin skin test (TST) result. The exact incidence of latent TB infection in Pakistani children is not known (21). The rate of TB case notification is 13% in children between 0 and 14 years of age per a 2019 WHO report (22). The rate of latent infection in children living with index TB cases ranges from 25% to 33% (23). We are unable to explain such low positivity levels in our cohort; however, the field staff who administered purified protein derivative (PPD) had formal training for administration of PPD and for reading the results.

Exploration of multiple host proteins as surrogates of TB disease is often used for the immunodiagnosis of TB (10) and monitoring of treatment responses (25). Several exploratory inflammatory (26) and metabolic (27) markers have been used to identify disease signatures among children. Among these, the use of serum biomarkers and CRP alone (28) or in combination with other proteins (24, 26) resulted in good accuracy in discriminating TB and monitoring responses after anti-TB treatment (25). We also observed CRP and ferritin as discriminatory serum biomarkers of disease in children compared to controls. Ferritin has been found to be present at elevated levels in TB patients in both adult (24, 29) and TB-exposed pediatric (30) cohorts. Elevated ferritin levels were shown to be associated with anemia of inflammation that resolved post-TB treatment (31). The presence of high ferritin and CRP levels in our cohort could be related to the nutritional status of TB cases or could represent an overt inflammatory process (32).

In conclusion, as a blood-based biomarker for TB disease, the MASC assay shows promise among children with microbiologically confirmed disease; however, the performance characteristics were suboptimal for the majority of young children with unconfirmed TB in this cohort.

## MATERIALS AND METHODS

### Study design and setting.

Participants were recruited from clinics affiliated with the Civil Hospital of Karachi; the Sindh Government Hospital, Korangi; and Aga Khan University Pediatric Primary Healthcare Centers (Rehri Goth and Bhains Colony locations). Using a case-control design, we evaluated the diagnostic accuracy of the MASC assay in distinguishing symptomatic children with TB disease from healthy controls.

### Study population.

Children presenting with pulmonary symptoms were consecutively approached by the study team of physicians (F. Arif, A. Mehnaz, and S. A. Siddiqi). Children were classified eligible as a “TB case” if they were between 1 and 14 years of age, had at least two symptoms consistent with pulmonary TB, and were residents within Karachi City and were ultimately treated for TB. Children were ineligible if they were currently receiving TB therapy, had received a BCG vaccine or tuberculin skin test (TST) within the past 8 weeks, or were immunosuppressed. Systematic evaluation for TB included a structured medical interview, anthropometric measurements, and radiologic evaluation; venous phlebotomy was conducted for study purposes, after which time a TST was performed and read within 48 to 72 h. For those who were able to produce a spontaneous sputum specimen, GeneXpert MTB/RIF (Cepheid, Sunnyvale, CA, USA) testing was performed. TB treatment was provided by their physicians through the NTP in accord with local guidelines (33, 34). All cases were reevaluated for study purposes 4 to 6 weeks after the start of TB therapy to verify symptom resolution, repeat anthropometry, and ensure tolerability of treatment.

Asymptomatic age-matched children were enrolled as controls, including children without known TB exposures or symptoms that could be consistent with TB disease in the previous 2 weeks. Children were ineligible if they had received TB therapy in the past, had received a BCG vaccine or had been subjected to a TST within the past 8 weeks, or had severe acute malnutrition. Controls underwent a structured medical interview, measurement of anthropometrics, and venous phlebotomy, after which time a TST was performed and read within 48 to 72 h. The caretakers of control participants received a brief education session on signs, symptoms, and prevention of TB via a flip chart. Similarly to the children with cases of TB, the control children had a follow-up visit 4 to 6 weeks after enrollment for clinical assessment and anthropometric measurements.

### Culturing of cells.

A single venous phlebotomy was conducted, and the volume of blood obtained was adjusted on the basis of age as follows: 3-ml volumes were collected from children <5 years of age, 6-ml volumes from children 5 to 9 years of age, and 12-ml volumes from children 10 to 14 years of age. Briefly, blood was mixed 1:1 with RPMI 1640 (Gibco, Grand Island, NY, USA) and layered on Histopaque (Sigma-Aldrich Chemie GmbH, Germany) for isolation of PBMCs. Live cells were counted and adjusted to a cell concentration of 5 or 10 million cells per ml in 10% fetal calf serum (FCS; Gibco), plated in 24-well tissue culture plates (Costar, Corning, USA), and incubated in the presence of 5% CO_2_. Incubation times depended on the concentration of PBMCs; 5 million PBMCs/ml were incubated for 48 and 72 h, while 10 million PBMCs/ml were incubated for 24 h per the micro-ALS (antibody in lymphocyte supernatant) method (5). Supernatants were divided into aliquots with protease inhibitor (Roche Diagnostics, Mannheim, Germany) and stored at –80°C for batch analysis.

### MASC ELISA for BCG-specific IgG responses.

The assay was performed as previously described (4, 5). Briefly, ELISA plates (Thermo Scientific) were coated with BCG antigen (1 μg/well) in coating buffer and incubated overnight before being washed with phosphate-buffered saline (PBS) and blocked with bovine serum albumin. Culture supernatant and positive/negative controls were run in duplicates; positive controls consisted of pooled sera from a well-characterized cohort of tuberculosis patients, and negative controls consisted of pooled sera from healthy controls from a setting of TB endemicity in Pakistan (35). PBS was used as a negative control. Plates were incubated for 2 h at 37°C. Secondary antibody (IgG) conjugated with horseradish peroxidase was added, and plates were incubated for 2 h with subsequent washing. Plates were finally developed using o-phenylenediamine tablets mixed in sodium perborate buffer (Sigma-Aldrich) and read at 490 nm using an ELISA reader (iMark absorbance reader; Bio-Rad, Hercules, CA). The absorbance was adjusted using “reagent blank” which contained BCG, conjugate, and substrate.

### Biomarker measurements.

Commercially available ELISA kits were used according to manufacturer’s instructions to measure biomarkers. High-sensitivity C-reactive protein (CRP, Calbiotech, Spring Valley, CA, USA) and ferritin (DiaMetra, Milan, Italy) levels were measured from undiluted plasma samples.

### Study definitions.

Cases were categorized as having confirmed TB, probable TB, or possible TB according to the initial NIH consensus criteria (36). Anthropometric measurements were performed using WHO AnthroPlus software (http://www.who.int/childgrowth/software/en/) for children (37). Z-scores for height for age (HAZ) were calculated as a marker of stunting, weight for height (WHZ) as a marker of wasting, and weight for age (WAZ) as a marker of chronic undernutrition; the latter two calculations included only those participants younger than 10 years. BMI for age (BAZ) was also calculated. Z-score categories were further defined as normal (Z-scores of greater than −2 standard deviations [SD]), moderate (Z-scores of less than or equal to −2 SD and greater than −3 SD), and severe (Z-scores of less than or equal to −3 SD). Stunting was defined as HAZ of less than or equal to −2 SD, wasting was defined as WHZ of less than or equal to −2 SD, malnutrition was defined as WAZ of less than or equal to −2 SD, and severe malnutrition was defined as HAZ of less than or equal to −3 SD.

### Statistical analysis.

Data were analyzed using the Statistical Package for Social Sciences (SPSS 15.0). All continuous variables are presented as means (± standard deviations) or medians, and categorical variables are presented as proportions where appropriate. Means were compared using *t* tests for two groups or analysis of variance (ANOVA) for more than two groups. Mann-Whitney and Kruskal-Wallis tests were applied for comparison of medians for two or more groups, respectively. Proportional differences were compared using Chi-square tests. Linear regression models were used to adjust for key characteristics, including age, gender, and nutritional status. A *P* value of <0.05 was considered statistically significant.

To understand the kinetics of IgG secretion against mycobacterial antigens, we compared median IgG responses and proportions of positive assays among participants who had MASC measurements obtained after 48 and 72 h of PBMC culture using the Mann-Whitney U test. The threshold for a positive assay for 48- and 72-h incubations was 0.35 optical density (OD) units, as previously published (8). IgG responses after 24 h of incubation (micro-ALS method) were also compared among groups. Since there are no validated thresholds for 24-h culture results among pediatric populations, a working threshold was devised by taking the mean MASC titer for control subjects and adding 3 standard deviations. Additionally, ROC analysis was carried out to determine the cutoffs at various time points using confirmed TB cases or all TB cases as the disease state variable.

### Ethics.

This protocol was approved by the Ethical Review Committee at Aga Khan University; the Institutional Review Board of Civil Hospital of Karachi, Dow University of Health Sciences; and the Institutional Review Board of the University of Virginia. Parents/caregivers of eligible children underwent the informed consent process and provided written parental permission for their child’s participation.
