# Supplementary figures and images for: Antibody-Secreting Cells To Diagnose Mycobacterium tuberculosis Infection in Children in Pakistan
Source: mSphere. 2020 Feb 5;5(1):e00632-19. doi: 10.1128/mSphere.00632-19 (PMC7002306; doi:10.1128/mSphere.00632-19)

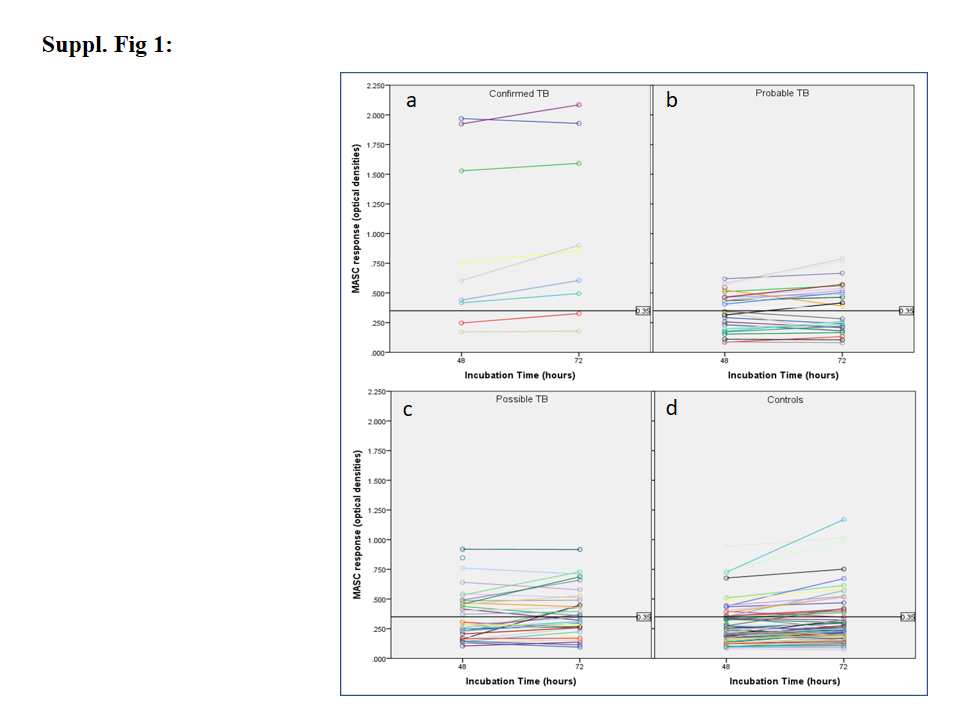

Supplement: FIG S1 [file mSphere.00632-19-sf001.tif]

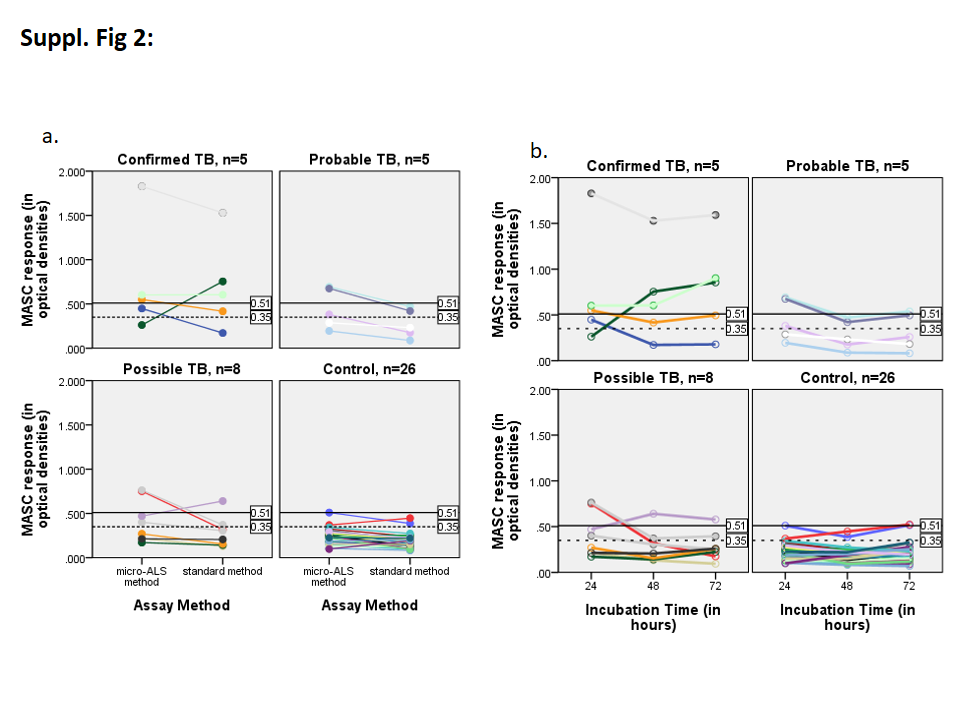

Supplement: FIG S2 [file mSphere.00632-19-sf002.tif]
