# Supplementary material for: Antibody-Secreting Cells To Diagnose Mycobacterium tuberculosis Infection in Children in Pakistan
Source: mSphere. 2020 Feb 5;5(1):e00632-19. doi: 10.1128/mSphere.00632-19 (PMC7002306; doi:10.1128/mSphere.00632-19)
Supplement: TABLE S1 [file mSphere.00632-19-st001.docx]

**Supplementary Table 1:**

| Concentration and incubation | <5 years | | | ≥ 5 years | | |
| --- | --- | --- | --- | --- | --- | --- |
|  | Cases n=18 | Controls n=18 | p-value | Cases n=61 | Controls n=57 | p-value |
| 24hour--10x10^6 | ---- | 0.28 ± 0.05  (n=2) | --- | 0.51 ± 0.38  (n=18) | 0.24 ± 0.09  (n=24) | **0.002** |
| 48hour --5x10^6 | 0.46 ± 0.41 | 0.38 ± 0.21 | 0.517 | 0.39 ± 0.31 | 0.24 ± 0.13 | **0.001** |
| 72hour--5x10^6 | 0.58 ± 0.51 (n=12) | 0.47 ± 0.31 (n=14) | 0.516 | 0.43 ± 0.32 | 0.28 ± 0.17  (n=53) | **0.004** |
