# Supplementary material for: Antibody-Secreting Cells To Diagnose Mycobacterium tuberculosis Infection in Children in Pakistan
Source: mSphere. 2020 Feb 5;5(1):e00632-19. doi: 10.1128/mSphere.00632-19 (PMC7002306; doi:10.1128/mSphere.00632-19)
Supplement: TABLE S2 [file mSphere.00632-19-st002.docx]

**Supplementary Table 2:**

|  |  | All cases vs. controls | | | All TB cases only | | | Microbiologically confirmed TB | | |
| --- | --- | --- | --- | --- | --- | --- | --- | --- | --- | --- |
|  |  | **5x10^6_48hrs** | | | **5x10^6_48hrs** | | | **5x10^6_48hrs** | | |
|  | **Model** | **Beta coeff** | **95% CI** | **P-value** | **Beta coeff** | **95% CI** | **P-value** | **Beta coeff** | **95% CI** | **P-value** |
| Age | Univariate | -0.009 | -0.022, 0.005 | 0.198 | -0.001 | -0.02, 0.02 | 0.920 | -0.123 | -0.25, 0.005 | 0.057 |
|  | Multivariate | -0.013 | -0.028, 0.002 | 0.096 | -0.003 | -0.03, 0.02 | 0.801 | -0.063 | -1.21, 1.08 | 0.885 |
| Gender | Univariate | -0.033 | -0.124, 0.057 | 0.471 | 0.008 | -0.15, 0.16 | 0.922 | 1.158 | -2.77, 0.46 | 0.134 |
|  | Multivariate | -0.011 | -0.108, 0.085 | 0.816 | 0.013 | -0.16, 0.19 | 0.882 | -0.071 | -7.13, 6.98 | 0.979 |
| HAZ | Univariate | -0.004 | -0.015, 0.006 | 0.409 | 0.004 | -0.04, 0.05 | 0.870 | -0.332 | -0.68, 0.017 | 0.059 |
|  | Multivariate | -0.007 | -0.017, 0.004 | 0.221 | 0.010 | -0.05, 0.07 | 0.802 | -0.170 | -2.01, 1.67 | 0.810 |
| BAZ | Univariate | -0.012 | -0.043, 0.020 | 0.468 | 0.0005 | -0.04, 0.05 | 0.979 | 0.130 | -0.18, 0.44 | 0.356 |
|  | Multivariate | -0.022 | -0.055, 0.011 | 0.189 | 0.002 | -0.05, 0.06 | 0.929 | -0.006 | -0.56, 0.55 | 0.979 |
