# Supplementary material for: Antibody-Secreting Cells To Diagnose Mycobacterium tuberculosis Infection in Children in Pakistan
Source: mSphere. 2020 Feb 5;5(1):e00632-19. doi: 10.1128/mSphere.00632-19 (PMC7002306; doi:10.1128/mSphere.00632-19)
Supplement: TABLE S3 [file mSphere.00632-19-st003.docx]

| **PBMCs concentration** | **All groups** | | **Confirmed TB** | | **Probable TB** | | **Possible TB** | | **Controls** | |
| --- | --- | --- | --- | --- | --- | --- | --- | --- | --- | --- |
|  | **rho** | **p value** | **rho** | **p value** | **rho** | **p value** | **rho** | **p value** | **rho** | **p value** |
| 10x10^6_24hrs | 0.302 | 0.093 | 0.70 | 0.188 | -0.20 | 0.800 | -0.243 | 0.599 | 0.362 | 0.168 |
| 5x10^6_48hrs | 0.237 | **0.012** | 0.410 | 0.273 | 0.584 | **0.004** | 0.077 | 0.685 | -0.002 | 0.989 |
| 5x10^6_72hrs | 0.215 | **0.029** | 0.351 | 0.354 | 0.378 | 0.110 | -0.087 | 0.660 | 0.067 | 0.656 |

**Supplementary Table 3:**
