# Supplementary material for: Antibody-Secreting Cells To Diagnose Mycobacterium tuberculosis Infection in Children in Pakistan
Source: mSphere. 2020 Feb 5;5(1):e00632-19. doi: 10.1128/mSphere.00632-19 (PMC7002306; doi:10.1128/mSphere.00632-19)
Supplement: TABLE S4 [file mSphere.00632-19-st004.docx]

**Supplementary Table 4:**

| **Growth difference** |  |  |  |  |  |
| --- | --- | --- | --- | --- | --- |
|  | **Indicator** | **Timeline** | **Mean ± sd** | **Mean difference ± sd** | **P-value** |
| Confirmed TB (n=9) | HAZ | Baseline | -1.98 ± 1.4 | -0.18 ± 0.28 | 0.083 |
|  |  | Follow up | -1.79 ± 1.15 |  |  |
|  | WAZ | Baseline | -3.07 ± 0.8 | -0.60 ± 0.69 | 0.271 |
|  |  | Follow up | -2.47 ± 0.87 |  |  |
|  | BAZ | Baseline | -2.29 ± 1.93 | -0.44 ± 0.70 | 0.097 |
|  |  | Follow up | -1.85 ± 1.73 |  |  |
| Probable TB (n=27) | HAZ | Baseline | -2.09 ± 1.77 | -0.71 ± 2.37 | 0.118 |
|  |  | Follow up | -1.38 ± 2.66 |  |  |
|  | WAZ | Baseline | -2.27 ± 0.92 | -0.45 ± 0.53 | 0.001 |
|  |  | Follow up | -1.82 ± 1.03 |  |  |
|  | BAZ | Baseline | -1.58 ± 1.74 | -0.04 ± 1.69 | 0.896 |
|  |  | Follow up | -1.54 ± 2.25 |  |  |
| Possible TB (n=29) | HAZ | Baseline | -2.30 ± 1.55 | -0.34 ± 0.93 | 0.027 |
|  |  | Follow up | -1.95 ± 1.81 |  |  |
|  | WAZ | Baseline | -2.26 ± 1.16 | -0.33 ± 0.57 | 0.002 |
|  |  | Follow up | -1.92 ± 0.96 |  |  |
|  | BAZ | Baseline | -1.32 ± 1.63 | -0.11 ± 1.08 | 0.503 |
|  |  | Follow up | -1.20 ± 1.59 |  |  |
| Controls | HAZ | Baseline | -0.34 ± 5.86 | 1.12 ± 5.92 | 0.114 |
|  |  | Follow up | -1.45 ± 1.13 |  |  |
|  | WAZ | Baseline | -1.04 ± 3.28 | 0.47 ± 3.42 | 0.341 |
|  |  | Follow up | -1.51 ± 0.87 |  |  |
|  | BAZ | Baseline | -0.97 ± 0.98 | 0.06 ± 0.57 | 0.352 |
|  |  | Follow up | -1.03 ± 0.96 |  |  |
